# On-demand pH-sensitive surface charge-switchable polymeric micelles for targeting *Pseudomonas aeruginosa* biofilms development

**DOI:** 10.1186/s12951-021-00845-0

**Published:** 2021-04-09

**Authors:** Xiangjun Chen, Rong Guo, Changrong Wang, Keke Li, Xinyu Jiang, Huayu He, Wei Hong

**Affiliations:** School of Pharmacy, Shandong New Drug Loading & Release Technology and Preparation Engineering Laboratory, Binzhou Medical University, 346 Guanhai Road, Yantai, 264003 People’s Republic of China

**Keywords:** Micelles, Biofilm penetration, Surface charge-switchable, Azithromycin

## Abstract

Bacterial biofilm is the complicated clinical issues, which usually results in bacterial resistance and reduce the therapeutic efficacy of antibiotics. Although micelles have been drawn attention in treatment of the biofilms, the micelles effectively permeate and retain in biofilms still facing a big challenge. In this study, we fabricated on-demand pH-sensitive surface charge-switchable azithromycin (AZM)-encapsulated micelles (denoted as AZM-SCSMs), aiming to act as therapeutic agent for treating *Pseudomonas aeruginosa* (*P. aeruginosa*) biofilms. The AZM-SCSMs was composed of poly(l-lactide)-polyetherimide-hyd-methoxy polyethylene glycol (PLA-PEI-hyd-mPEG). It was noteworthy that the pH-sensitive acylhydrazone bond could be cleaved in acidic biofilm microenvironment, releasing the secondary AZM-loaded cationic micelles based on PLA-PEI (AZM-SCMs) without destroying the micellar integrity, which could tailor drug-bacterium interaction using micelles through electrostatic attraction. The results proved that positively charged AZM-SCMs could facilitate the enhanced penetration and retention inside biofilms, improved binding affinity with bacterial membrane, and added drug internalization, thus characterized as potential anti-biofilm agent. The excellent in vivo therapeutic performance of AZM-SCSMs was confirmed by the targeting delivery to the infected tissue and reduced bacterial burden in the abscess-bearing mice model. This study not only developed a novel method for construction non-depolymerized pH-sensitive SCSMs, but also provided an effective means for the treatment of biofilm-related infections.

**Supplementary Information:**

The online version contains supplementary material available at 10.1186/s12951-021-00845-0.

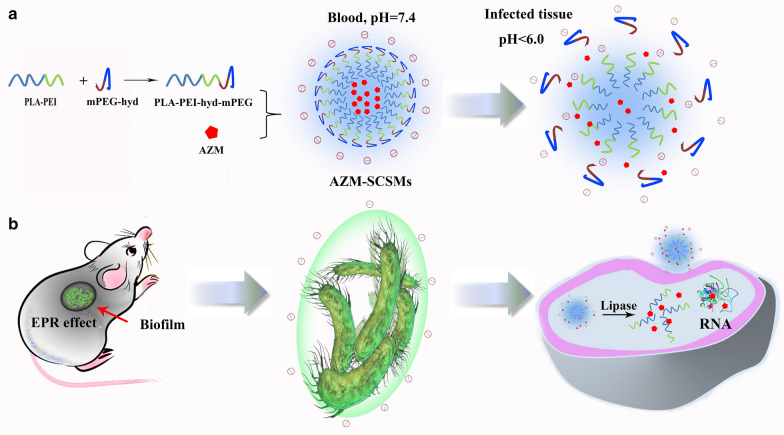

## Introduction

Bacterial infection could cause serious diseases or even death, and has caused sustained extensive concerns in the past few decades [1, 2]. Unfortunately, this situation becomes even worse when confronting biofilm [3, 4]. Biofilms can act as an dependable physical and metabolic barrier, restricting antibiotics penetration and inducing antibiotics inactivation [5]. *Pseudomonas aeruginosa* (*P. aeruginosa*) is one of the clinical biofilm-forming bacteria, which can easily colonize and form biofilms in the airways of cystic fibrosis (CF) patients to prevent antibiotics penetration [3, 6–9]. Thus, it is necessary to develop a more effective drug delivery system to combat the increased bacterial infection especially in biofilm.

Cationic polymeric micelles have recently showed its potential to solve this problem [10–17]. In virtue of the negatively charged bacterial cells, many kinds of polymeric micelles have been designed for the therapy of biofilms achieving the effective penetration and retention inside the biofilm [18–22]. Nevertheless, they are rapidly discovered by host immune cells and have a short blood circulation time [23, 24]. Based on the current situation, it is an urgent problem to attain simultaneous long circulation time and enhanced biofilm penetration by one antibiotic nano-delivery system. By means of the acidic biofilm microenvironment, pH-sensitive surface charge-switchable micelles were prepared for biofilm treatment [25–27]. Shi et al. designed pH-sensitive mixed-shell polymeric micelles (MSPMs) based on poly(ethylene glycol)-poly(ε-caprolactone) (PEG-PCL) and poly(β-amino ester)- poly(ε-caprolactone) (PAE-PCL). MSPMs could response to the acidic biofilm microenvironment and switch their surface charge from negative to positive, consequently, sticked firmly to the bacterial membrane and prevented rapid wash-out. MSPMs would effectively kill bacteria in their biofilm mode of growth [25]. Thus, the elaborate control of surface charge by biofilm microenvironment for improved penetration and retention may stand for a potential strategy for biofilm-associated infections in the future.

**Fig. 1:**
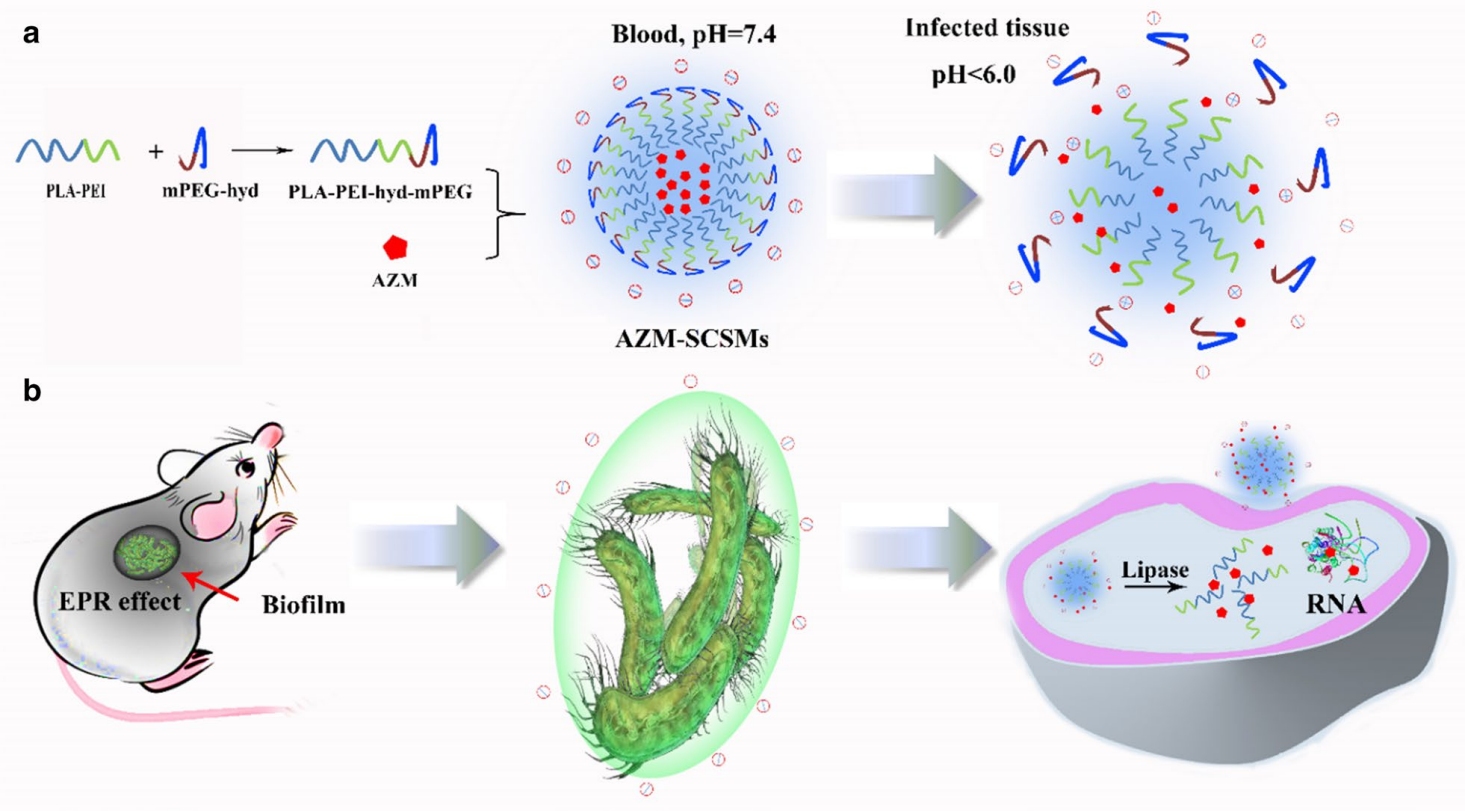
Summary of the AZM-loaded surface charge-switchable micelles (AZM-SCSMs) under the influence of pH changes (**a**). The mechanism for targeting delivery of encapsulated drug into bacteria deep into biofilms for treatment biofilm-related infections in vivo (**b**)

Most previous studies employed pH-sensitive block to construct surface charge-switchable micelles. However, the protonation of pH-sensitive block was usually accompanied with the dissociation of micellar structure. Retaining the integrity of micellar structure at acidic pH values was important to tailor drug-bacterium interactions and biofilm penetration. Herein, in this research, we induced acylhydrazone bond to construct pH-sensitive surface charge-switchable polymeric micelles (SCSMs) based on PLA-PEI-hyd-mPEG (Fig. 1). Similar as previously reported SCSMs, our acylhydrazone bonds based SCSMs could also reduce rapid clearance by the mononuclear phagocytic system due to the PEG shell and target areas of infection through localized increases in vascular permeability. After initial penetration into a biofilm, the advantages of our SCSMs became evident. Firstly, the acylhydrazone bonds would preferentially peel off once pH decreasing and immediately switch the surface charge to a positive one. Thus, even a relatively short residence time at an acidic site of infection might be sufficient to enable binding to bacteria. Secondly, the breakage of acylhydrazone bonds would not cause the disassociation of the micellar structure. This was important in that it would retain the slow releasing characteristics of intact micelles and tailor drug-bacterium interactions using micelles. Finally, the released secondary micelles (SCMs) based on PLA-PEI could target themselves firmly to negatively charged bacterial surfaces to prevent rapid wash-out and promote bacterial membrane permeability to assist the internalization of cargoes into bacteria. To prove the above hypotheses, we evaluated the pH-dependent physicochemical properties of SCSMs and the micelles-bacterium binding affinity through zeta potential analysis, fluorescence microscope observation, flow cytometry assay and bio-layer interferometry. Then, SCSMs were subsequently loaded with azithromycin, and the antibacterial efficiency was investigated at the cellular and animal levels. Results confirmed that AZM-SCSMs exhibited outstanding therapeutic effect against *P. aeruginosa*-induced biofilms both in vitro and in vivo.

### Materials and methods

#### Materials

#### Reagents

The copolymers of PLA_5K_-PEI_2K_-mPEG_5K_ and PLA_5K_-PEI_2K_-hyd-mPEG_5K_ were purchased from Ruixi Biological Technology Co., Ltd (Xi’an, Shanxi, China). The details of synthesis and characterization of the copolymers were shown in the Additional file 1. Biotin LPS and A LIVE/DEAD® *Bac*Light™ Bacterial Viability Kit were obtained from Nanocs, Inc. (New York, USA) and Thermo Fisher Scientific Inc. (Shanghai, China), respectively. Cyanine 5.5 (Cy 5.5) and azithromycin (AZM) were obtained from Macklin Biochemical Co., Ltd (Shanghai, China).

#### Bacteria and cells

*Pseudomonas aeruginosa* (*P*. *aeruginosa*) ATCC9027 was purchased from American Type Culture Collection (Manassas, VA, USA), which were maintained in 30% glycerol at -80 ºC until use. VERO cells were also purchased from American Type Culture Collection (Manassas, VA, USA), and culture in DMEM media (Gibco BRL, Grand Island, NY) containing 10% FBS.

#### Animals

Male BALB/c nude mice and BALB/c mice were used for the in vivo biodistribution study and the in vivo therapeutic treatment of biofilm infection, respectively. The animals were obtained from Pengyue Laboratory Animal Breeding Co., Ltd (Jinan, Shandong, China) and the animal studies were conducted according to the experimental protocols by Institutional Animal Care and Use Committee of Binzhou Medical University.

#### Micellar formulations

SCSMs: blank surface charge-switchable polymeric micelles composed of PLA-PEI-hyd-mPEG;

SCUMs: blank surface charge-unswitchable polymeric micelles composed of PLA-PEI-mPEG;

SCMs: blank secondary cationic micelles composed of PLA-PEI.

AZM-SCSMs: SCSMs loaded with AZM;

AZM-SCUMs: SCUMs loaded with AZM;

AZM-SCMs: SCMs loaded with AZM;

Cy5.5-SCSMs: SCSMs loaded with Cy5.5;

Cy5.5-SCUMs: SCUMs loaded with Cy5.5;

Cy5.5-SCMs: SCMs loaded with Cy5.5;

### Methods

#### Preparation and characterization of polymeric micelles

The SCSMs suspensions were prepared by the thin-film hydration method [28]. Briefly, 180 mg of PLA-PEI-hyd-mPEG was dissolved in 20 mL mixed solvent of dichloromethane and methanol (volume ratio 1:1). The film was obtained by rotary evaporation of the solvent. Then, the film was hydrated with 20 mL PBS, and followed by filtration through a 0.22 μm film. SCUMs were prepared with the same procedure except using PLA-PEI-mPEG as copolymers. The AZM-SCSMs and AZM-SCUMs were prepared by encapsulating 20 mg of AZM. The micelles morphology was investigated on a JEM1400 TEM (JEOL, Japan), and the particle size and zeta potential were measured on a Zetasizer Nano ZS analyzer (Malvern, UK). Drug loading coefficient (DL%) and entrapment efficiency (EE%) were calculated by Eqs. (1) and (2), respectively:

1 $$DL\%=\frac{\text{Weight of the drug in micelles}}{\text{Weight of the feeding copolymers and drug}}\times 100\%$$

2 $$EE\%=\frac{\text{Weight of the drug in micelles}}{\text{Weight of the feeding drug}}\times 100\%$$

A dialysis bag (WM, 12–14 kDa) containing 2 mL freshly prepared AZM-SCSMs and AZM-SCUMs was incubated in 20 mL PBS (10 mM, pH 5.5 or pH 7.4), and aliquots of the dialysis solution were collected at predetermined time intervals, colored with 75% sulfate solution, and then subjected to absorbance measurement at 482 nm on a Synergy H1 microplate reader (Biotek Instruments, Inc., USA).

#### pH-dependent physicochemical properties

The changes of morphology, particle size and zeta potential of SCSMs and SCUMs along with pH decrease over time were investigated on a JEM1400 transmission electron microscopy (TEM, JEOL, Japan) and a Zetasizer Nano ZS analyzer (Malvern, UK), respectively.

#### Micelles-bacterium binding study

##### Zeta potential analysis

The planktonic micelles-bacterium binding study was initialized by adding 10 mL of the tested blank micelles (SCSMs and SCUMs) to 10 mL of bacterial suspensions (10^8^ CFU) with pH values adjusted to 7.4 and 5.5, respectively. The zeta potential of the micelles/bacteria mixture was measured on a Zetasizer Nano ZS analyzer (Malvern, UK) for each solution at predetermined time points.

##### Fluorescence microscope (FM)

The bacteria were suspended in PBS at pH of 5.5 or 7.4, and incubated with Cy5.5-SCSMs or Cy5.5-SCUMs solutions, respectively. As scheduled time points (0.5 h, 2 h, 4 h, 8 h, 12 h and 24 h), the bacteria solution was washed twice with PBS to remove the unbound micelles, and then resuspended in 100 μL PBS. The red fluorescent microscope images were captured by Olympus BX53F2 Optical Microscope (Olympus Corporation, Tokyo 163–0914, Japan).

##### Flow cytometry assays

The bacterial suspensions were pretreated as described above in the FM section, and then BD FACSCanto II flow cytometry (USA) was used to acquire red fluorescence data. The untreated negative sample was used as control.

##### Bio-Layer Interferometry (BLI)

The BLI study was performed on Octet RED 96e (ForteBio, USA). The pH of blank SCSMs and SCUMs was firstly adjusted to 7.4 and 5.5, respectively. The biotin-linked lipopolysaccharide (b-LPS) was loaded on streptavidin (SA) biosensor. Association and dissociation experiments were conducted for 90 and 120 s, respectively.

#### Time-dependent biofilm penetration

The mature biofilms were established as previously reported [29], and the petri dishes with biofilms attached were washed twice with saline, before incubated in PBS (pH 7.4 and 5.5) containing Cy5.5-SCSMs or Cy5.5-SCUMs for 0.5 h, 1 h, 2 h, 4 h and 8 h, respectively. The biofilm images were obtained on a Zeiss LSM 880 (Zeiss, Germany) after stained with SYTO 9 dye solution for 30 min. The Z-stack imaging was carried out using the areas near the center of the dishes at a 1-μm interval.

#### In vitro antibacterial activity against planktonic bacteria

##### Minimum inhibitory concentration

The minimal inhibitory concentrations (MICs) of free AZM or AZM-loaded micelles (AZM-SCSMs and AZM-SCUMs) were determined against *P*. *aeruginosa* ATCC9027 under pH 7.4 or 5.5 by a micro-dilution method, respectively [30, 31].

##### Scanning electron microscopy (SEM) observation

SEM observation was made with the same procedure as our previous study [32]. Briefly, the exponential phase *P*. *aeruginosa* ATCC9027 strain (ca. 10^7^ CFU/mL) was incubated with 8 μg/mL of different formulations (Negative Control, free AZM, AZM-SCSMs and AZM-SCUMs) for 12 h under different pHs (7.4 or 5.5). After incubation, the cells were harvested, fixed, and then gradient dehydrated. Finally, the cells were dropped on polylysine-coated slides, dried, coated with gold before examination through a SEM (Zeiss EVO LS15, Oberkochen, Germany).

#### Biofilm susceptibility

The effects of free AZM or AZM-loaded micelles on the mature biofilms of *P*. *aeruginosa* were investigated by CLSM. The biofilms were incubated with free AZM or AZM-loaded micelle (16 to 512 µg/mL, pH 7.4 or 5.5) for 24 h, and then stained with a LIVE/DEAD BacLight Bacterial Viability Kit at 25 °C for 30 min. The residual biofilm images were obtained through a Zeiss LSM 880 microscopy (Zeiss, Germany). The Z-stack imaging was conducted using the areas near the center of the dishes at a 1-μm interval.

#### In vitro cytotoxicity and hemolysis assay

The cytotoxicity of AZM-SCSMs was tested on Vero. Briefly, the cells were seeded in 96-cell plates at a density of 3000 cells per well for 24 h, followed by treatment with different concentrations of AZM-SCSMs (with a final AZM concentration of 125 ~ 2000 μg/mL) for another 24 h. The cell viability was determined using the MTT assay, as described preciously [28].

The hemolysis assay was performed on the AZM-SCSMs against rabbit red blood cells (rRBCs) as descried previously [33]. Briefly, a total of 200 µL rRBC (1%, v/v) solution was incubated with 200 µL AZM-SCSMs (with a final AZM concentration of 125 ~ 2000 μg/mL) at 37 °C for 2 h and then centrifuged at 1000*g* at 4 °C for 5 min. The supernatant was transferred to 96-well plates, and the optical density at 576 nm was measured using a Synergy H1 hybrid multi-mode microplate reader (BioTek Instruments, Inc., USA) to monitor the release of hemoglobin. The negative and positive controls were rRBCs in saline and 0.5% Triton X-100, respectively. The release of hemolysis was measured using Eq. (3):

3 $$\text{Hemolysis (\%})=\left[\left({OD}_{t}-{OD}_{0}\right)/\left({OD}_{100}-{OD}_{0}\right)\right]\times 100\%$$

where *OD*_*t*_ is the absorption of rRBCs in AZM-SCSMs at the concentration of t, *OD*_*0*_ is the absorption of rRBCs in saline, and *OD*_*100*_ is the absorption of rRBCs in 0.5% Triton X-100.

#### In vivo biodistribution study

In vivo biodistribution was measured using an infected mouse model established as previously reported [32, 34]. *P*. *aeruginosa* (250 μL, 10^8^ CFU) was subcutaneously administered into the back of male BALB/c nude mice. On day two after infection, an obvious infected abscess had arisen subcutaneously in each test nude mouse. Then, 200 µL of Cy5.5-SCSMs and Cy5.5-SCUMs was injected into the abscess-bearing nude mice via tail vein and the near-infrared fluorescence imaging was measured at 1 h, 2 h, 4 h, 8 h, 12 h, 24 h, 36 h and 48 h on IVIS® Spectrum (PerkinEImer, USA).

#### In vivo therapeutic effect against *P*.* aeruginosa* biofilm

To access the in vivo anti-biofilm effect of AZM-SCSMs, a subcutaneous abscess was experimentally carried out in each tested BALB/c mouse as described above. On day two after infection, a total of 25 mg/kg free AZM, AZM-SCSMs and AZM-SCUMs were intravenously injected once daily for 3 days (n = 7). Sterile saline was injected as the control. After the therapy, these abscesses were harvested and analysis by standard plate count methods.

##### Statistical Analyses

The data were expressed as mean ± standard deviation (S.D). Student’s *t-test* was performed to evaluate the difference between two groups. Statistical significance was defined as ^*^P < 0.05, ^**^P < 0.01, ^***^P < 0.001 and ^****^P < 0.0001.

## Results

### Characterization of AZM-loaded polymeric micelles

**Fig. 2:**
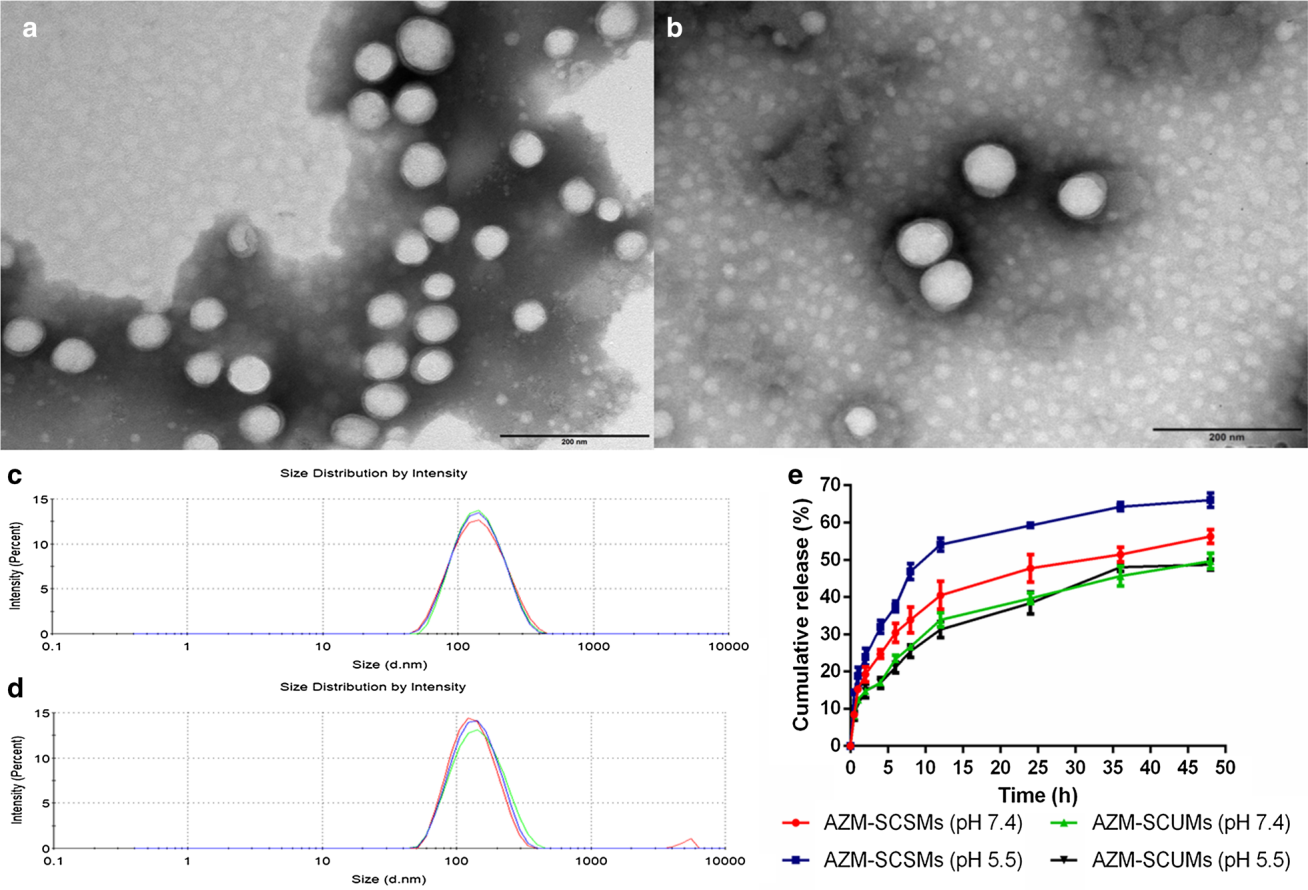
The morphology images of AZM-SCSMs (**a**) and AZM-SCUMs (**b**) obtained by TEM (N = 3). Scale bars = 100 nm. The particle size and distribution of AZM-SCSMs (**c**) and AZM-SCUMs (**d**) measured by DLS. The cumulative release of AZM from AZM-SCSMs and AZM-SCUMs at pH 7.4 or pH 5.5, respectively (**e**)

The characteristics of the micelles at pH 7.4 were summarized in Table 1. Both blank SCSMs and SCUMs had a similar diameter around 80 nm, while the diameters increased to 120 nm after loading with AZM, possibly because of the relatively high drug loading coefficient (nearly 80%). Both AZM-SCSMs and AZM-SCUMs were slightly negatively charged at pH 7.4, the almost net surface charge could avoid being recognized by opsonin and passively target the infection sites [35]. A spherical and homogeneous morphology was observed by TEM (Fig. 2a, b), and the particle size was consistent with that obtained by DLS (Fig. 2c, d).

**Table 1:** The physicochemical characteristics of different micellar formulations (n = 3)

Formulations	Particle size (nm)	Zeta potential (mv)	PDI	DL%	EE%
AZM-SCSMs	121.7 ± 1.6	− 0.34 ± 0.053	0.210 ± 0.014	7.94 ± 0.12	78.97 ± 0.99
AZM-SCUMs	123.2 ± 4.2	− 0.41 ± 0.075	0.221 ± 0.021	7.91 ± 0.069	79.16 ± 0.38

The releasing behaviors of AZM from tested micelles under pH 7.4 and 5.5 were evaluated, respectively. As shown in Fig. 2e, SCSMs showed pH-dependent release behavior, a 10% increase in release corresponded with the total loading was observed under pH 5.5 than 7.4. It could be due to the breakage of acylhydrazone bond under acidic condition disrupting the hydrophobic/hydrophilic balances, accelerating the releasing behavior. However, the cleavage of acylhydrazone bond did not cause burst release. The drug release from AZM-SCUMs was characterized as a burst releasing at initial followed by a sustained release pattern lasting for 48 h with approximately 50% of the total loaded AZM released, and remained basically unchanged at pH 7.4 and 5.5,

### pH-dependent physicochemical properties of micelles

**Fig. 3:**
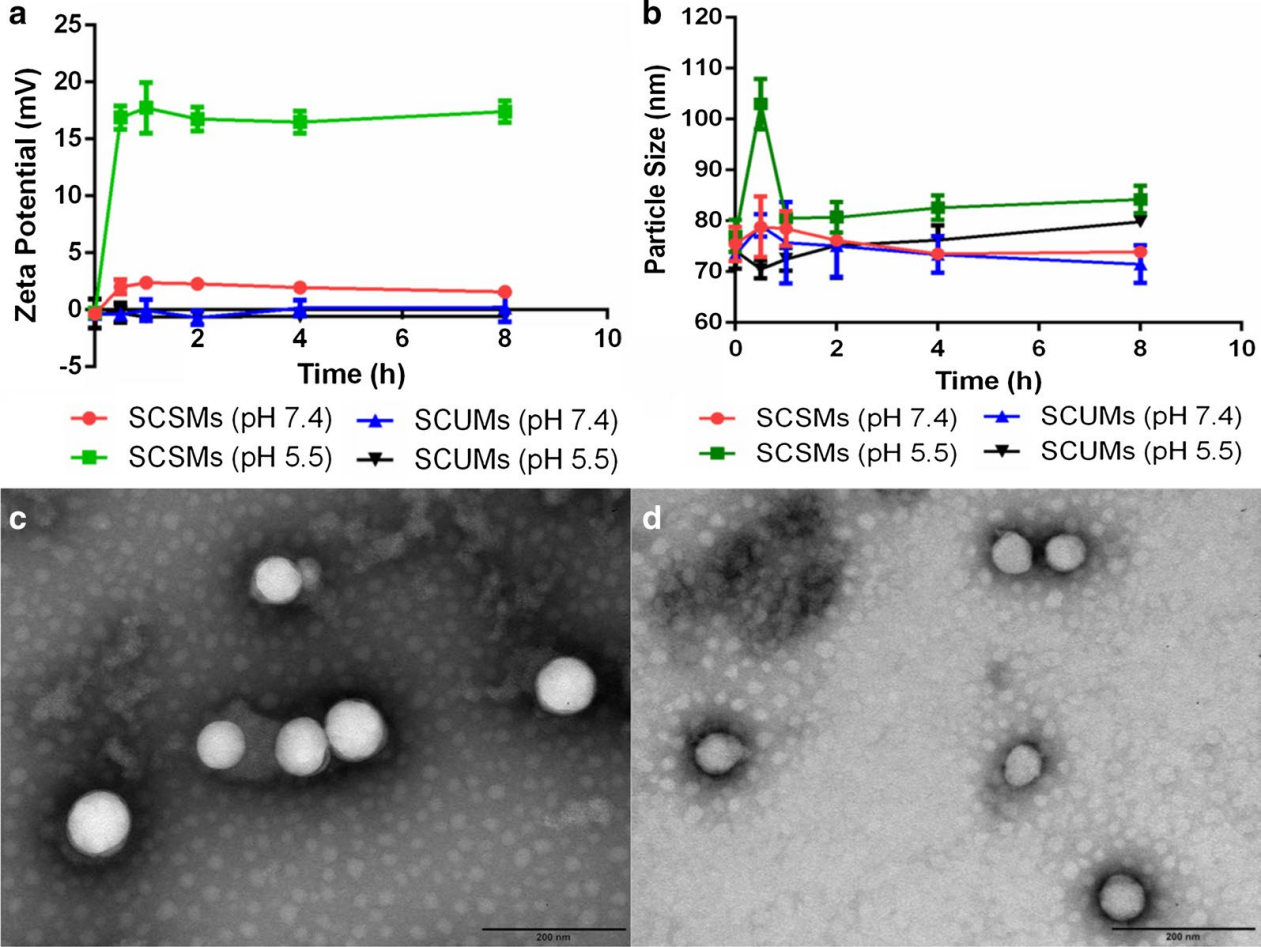
The variations in Zeta potentials (**a**) and particle sizes (**b**) of SCSMs and SCUMs vs time under pH 7.4 and 5.5, respectively. Error bars denote the standard deviations. TEM images of SCSMs after incubation at pH 5.5 for 0.5 h (**c**) and 8 h (**d**). Scale bars = 200 nm. n = 3 for all research

The physicochemical properties of SCSMs as a function of pH were evaluated. The particle size and zeta potential were firstly evaluated by DLS (Fig. 3a, b). The surface charge of SCSMs could quickly switched to a positive one (≈ + 18 mv) at pH 5.5 within 0.5 h, suggesting that even a relatively short stay time at an acidic site of infection might be sufficient to achieve positive charge reversal and attraction. At pH 7.4, a subtle increase also occurred, which might be due to the partial cleavage of acylhydrazone bond at pH 7.4. However, this positive charge reversal could not increase the phagocytic uptake and affect biodistribution of SCSMs during the circulation time. Previous study indicated that keeping the zeta potential below 15 mv could effectively escape the phagocytic uptake [24]. In contrast, SCUMs were insensitive to pH, and the zeta potential kept negatively charged at both pHs. SCSMs showed an increase of particle size around 20 nm within 0.5 h after incubation at pH 5.5, but the particle size returned to 80 nm again after 1 h of incubation and kept stable with the incubation time extending to 8 h. The fluctuation of particle size may be due to the corresponding change of the micellar structure under acidic condition. At the physiological pH, PLA-PEI-hyd-mPEG could form core–shell structure taking PLA as the inner core and PEI/mPEG as the outer shell with the particle size around 80 nm. Along with pH decreasing, acylhydrazone bond began to hydrolyze, the hydrophilic/hydrophobic balance was disturbed which led to the increase of micelle sizes. After hydrolysis completion, the secondary micelles basing on PLA as the inner core and PEI as the outer shell were formed with similar particle size around 80 nm. The size of SCUMs demonstrated little differences with decreasing pH. Additionally, TEM images confirmed the pH-triggered size increase and ability to retain micellar integrity of SCSMs at acidic pH values after incubation of 0.5 h (Fig. 3c) and 8 h (Fig. 3d), suggesting that the SCSMs could enable drug-bacterium interactions at the site of infection.

### pH-dependent bacterial targeting and biofilm penetration

**Fig. 4:**
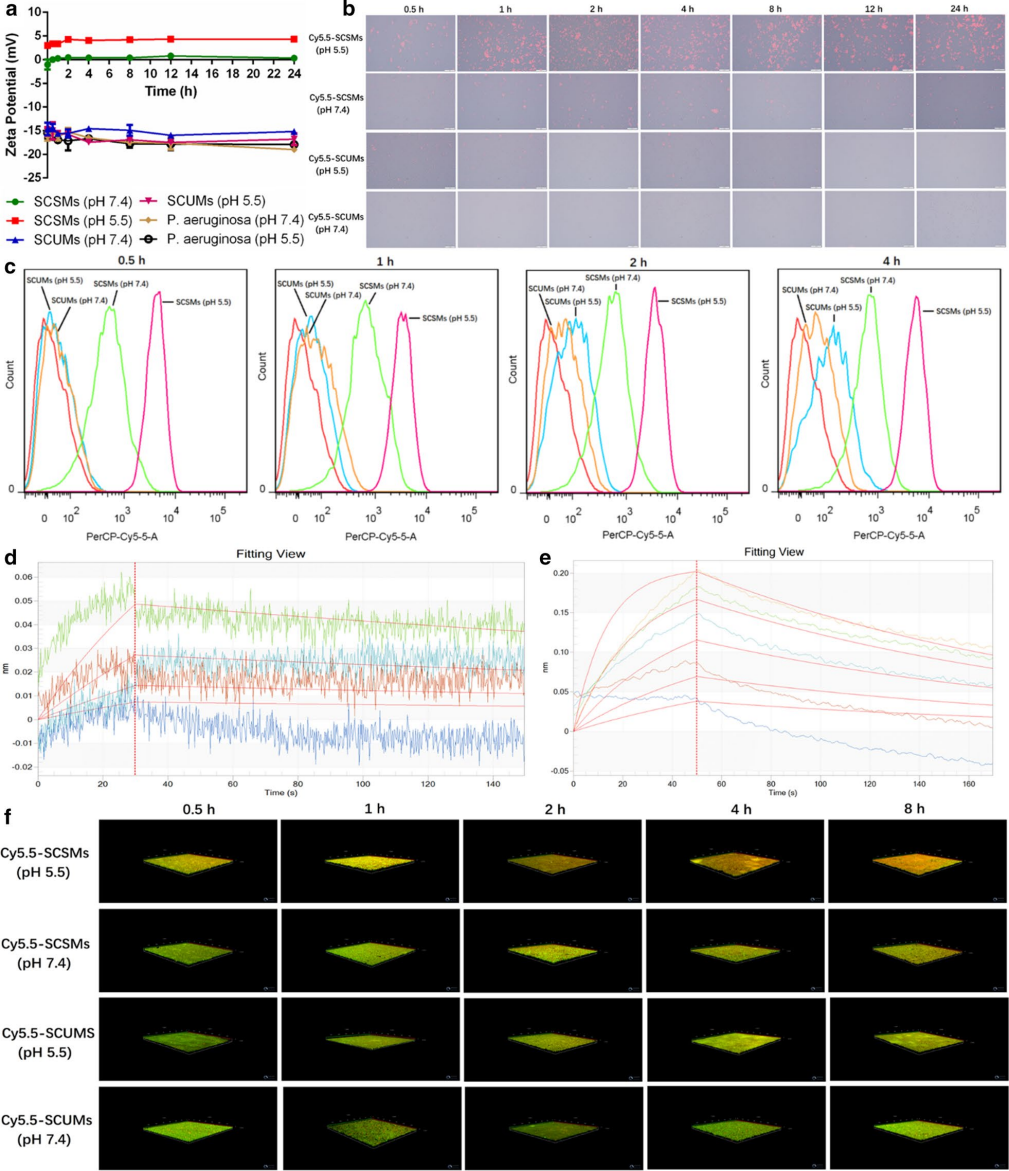
Zeta potential changes of *P. aeruginosa* after incubation with SCSMs and SCUMs under pH 7.4 and 5.5 for different periods of time (0–24 h) (**a**). Qualitative (**b**) and quantitative (**c**) cellular uptake of *P. aeruginosa* incubated with Cy5.5-SCSMs and Cy5.5-SCUMs for different periods of time (0.5–24 h) under pH 7.4 and 5.5. BLI assay for the interaction between b-LPS and SCSMs under pH 7.4 (**d**) and 5.5 (**e**), respectively. pH-dependent penetration and bacterial targeting of Cy5.5 loaded micelles in *P. aeruginosa* biofilms at pH 7.4 and 5.5, respectively (**f**)

We evaluated whether the observed surface charge change of SCSMs with pH would enable differential binding to bacteria. The micelles-bacteria binding behavior under different pH (5.5 and 7.4) was initially investigated by zeta potential analysis (Fig. 4a). *P. aeruginosa* could maintain stable and anionic charge during the investigation time under both pHs, which would yield conservative confirmation of our zeta potential-based results. The zeta potentials of the bacteria after incubation with SCSMs were increasingly more positive when the pH dropped to 5.5, suggesting that pH induced surface charge positive reversal could tailor more micelles targeted toward the negatively charged bacterial cell surfaces. No obvious zeta potential changes were observed for *P. aeruginosa* treated with SCUMs under pH 7.4 and 5.5, demonstrating that the interaction between micelles and bacteria was limited and pH-insensitive. Next, we performed visual confirmation of pH-dependent binding behavior of SCSMs to bacteria using fluorescence microscopy. As shown in Fig. 4b, for Cy5.5-SCSMs treatment, strong red fluorescence could be seen at pH 5.5 but not pH 7.4. Moreover, agglutination of bacteria was also observed in pH 5.5 Cy5.5-SCSMs treated group. The main reason for the result is the positively charged SCMs bridging two or more negatively charged bacteria together. On the contrary, the bacteria interaction with SCUMs remained weak characterized as invisible red fluorescence despite of the pH variation. Then, we executed a kinetic research of SCSMs binding to *P. aeruginosa*, and the bacteria-related fluorescence was measured via flow cytometry. In line with the FM observation, a large amount of SCSMs was taken up by *P. aeruginosa* (Fig. 4c) at pH 5.5. Additionally, ~ 80% of maximal binding occurred within 0.5 h, which suggested that even the shorter residence time at an acidic site of infection is enough to facilitate the binding between bacteria and SCSMs. Lipopolysaccharide (LPS) is a main surface component of Gram-negative bacteria, negatively charged. Thus, the interactions between SCSMs and LPS under different pHs were quantitatively monitored by BLI (Fig. 4d). SCSMs exhibited a pH-dependent association pattern with LPS, which showed less affinity with LPS under physiological pH, but stronger interaction under acidic condition, with the affinity constants K_D_ of 1.27 × 10^–7^ M and 4.40 × 10^–9^ M (Table 2), respectively. When pH value is 5.5, acylhydrazone bond would be dissociated and then secondary cationic PLA-PEI micelles (SCMs) were discharged, leading to the adhesion onto negatively charged LPS through electrostatic interaction. In contrast, without releasing of SCMs, SCSMs could hardly attached to LPS due to electrostatic repulsion.

**Table 2:** Binding parameters of b-LPS with SCSMs under different pHs measured by BLI

Analyte	k_on_ (1/Ms)	k_dis_ (1/s)	K_D_ (M)
SCSMs (pH 7.4)	4.09E+04	5.00E−03	1.23E−07
SCSMs (pH 5.5)	1.40E+06	6.18E−03	4.40E−09

Next, to prove stealth penetration and accumulation in biofilms, green-fluorescent biofilms were exposed to Cy5.5 loaded micelles and imaged through CLSM (Fig. 4f). Perhaps due to the absence of interaction with the bacteria in the biofilms, the limited penetration and accumulation at neither pH 7.4 nor pH 5.5 were observed. Nevertheless, SCSMs demonstrated a quite various pattern. CLSM results indicated no obvious red-fluorescent of SCSMs at pH 7.4, Cy5.5-SCSMs permeated well into the biofilms at pH 5.5, even up to the bottom of the biofilm. Obviously, due to the active interaction of SCSMs with bacterial cell surfaces allowed the gathering of abundant concentrations of Cy5.5-SCSMs in a weak acid environment in comparation with SCUMs. Thus, SCSMs could achieve biofilm-specific targeting through a combination of weaker non-specific binding in the blood but avid bacterial binding when arrived in acidity-related biofilms infections.

### Effect of AZM-loaded micelles on the growth of planktonic and biofilm bacteria

The MIC values of free AZM and AZM-loaded micelles were determined and listed in Table 3. At pH 7.4, free AZM was the most effective formulation of the drug, but that was declined with pH. At pH 5.5, free AZM lost potency by a factor of 4.0. AZM-SCUMs needed higher initial AZM concentration compared with free drug to reach antibacterial effects at physiological pH 7.4, and similarly proved pH-sensitive loss in activity by a factor of 2. As expected, AZM-SCSMs behaved similarly to AZM-SCUMs at pH 7.4, but had obviously enhanced activity at pH 5.5, with the MIC of 8 μg/mL.

**Table 3:** Antibacterial activities of the tested formulations

Formulations	MIC (µg/mL)		
	AZM	AZM-SCSMs	AZM-SCUMs
pH 7.4	8	64	64
pH 5.5	32	8	128

**Fig. 5:**
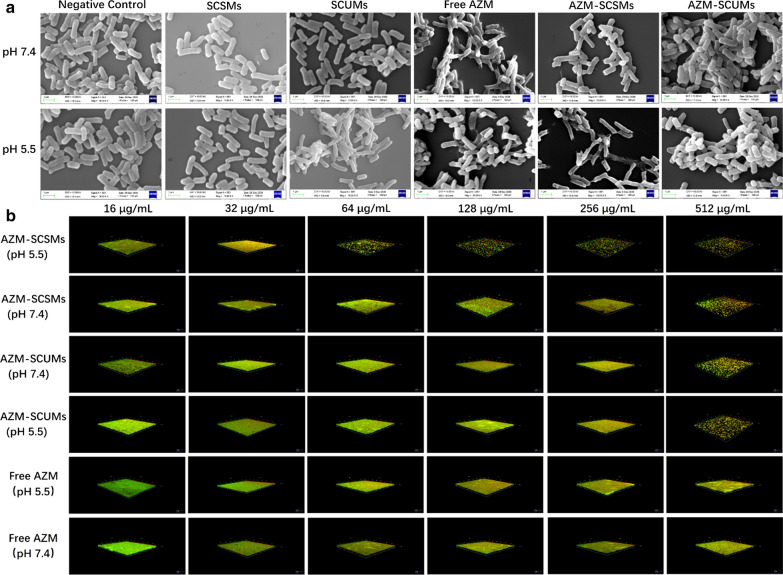
SEM micrographs of *P. aeruginosa* treated by free AZM, AZM-SCSMs and AZM-SCUMs under pH 7.4 and 5.5 for 12 h (**a**). CLSM analysis of *P. aeruginosa* biofilm eradication (**b**). The *P. aeruginosa* biofilms were treated with free AZM, AZM-SCSMs and AZM-SCUMs under pH 7.4 and 5.5 for 24 h at different concentrations

The morphological changes of bacteria before after treatment with different formulations at a dose of 8 μg/mL were further investigated through SEM. As shown in Fig. 5a, untreated *P. aeruginosa* exhibited a smooth surface at both pHs. At pH 7.4, there was no obvious cell membrane damage was observed except the bacteria treated with free AZM. Most of the bacteria shrank, with the appearance of membrane distortion, blisters, and breakage, and the leaked protoplasm from the bacteria manifested viscidity and enhanced aggregation. In sharp contrast, at pH 5.5, free AZM lost its antibacterial activity, the cells surface became rough after incubation with AZM-SCSMs and lysis of the cells occurred. This suggested that facilitating micelles-bacterium interactions under acidic conditions could not only enhance the antibacterial activity, but also partially mitigate the loss of activity with pH, further highlighting the potential of this delivery system for treating infections associated with localized acidity.

AZM has aroused wide attention due to effective therapy effects by inhibition the growth of biofilms via inhibiting quorum-sensing [36]. Nevertheless, for the already formed biofilms, the therapeutic efficacy of AZM behaved badly. Thus, the anti-biofilm efficacy of AZM-SCSMs was investigated against *P. aeruginosa* in their biofilm mode of growth. Carried out the experiment as a function of concentration, we turned to CLSM (Fig. 5b). At pH 5.5, the advantage with respect to a reduction in viability of *P. aeruginosa* biofilm of AZM-SCSMs became evident at a low concentration of around 32 μg/mL. However, at pH 7.4, AZM-SCSMs almost lost anti-biofilm potency and required higher AZM concentration (512 μg/mL) to eradicate biofilm. Both free AZM and AZM-SCUMs showed limited anti-biofilm effects among the tested concentrations at both pHs.

### In vitro cytotoxicity and hemolysis assays

**Fig. 6:**
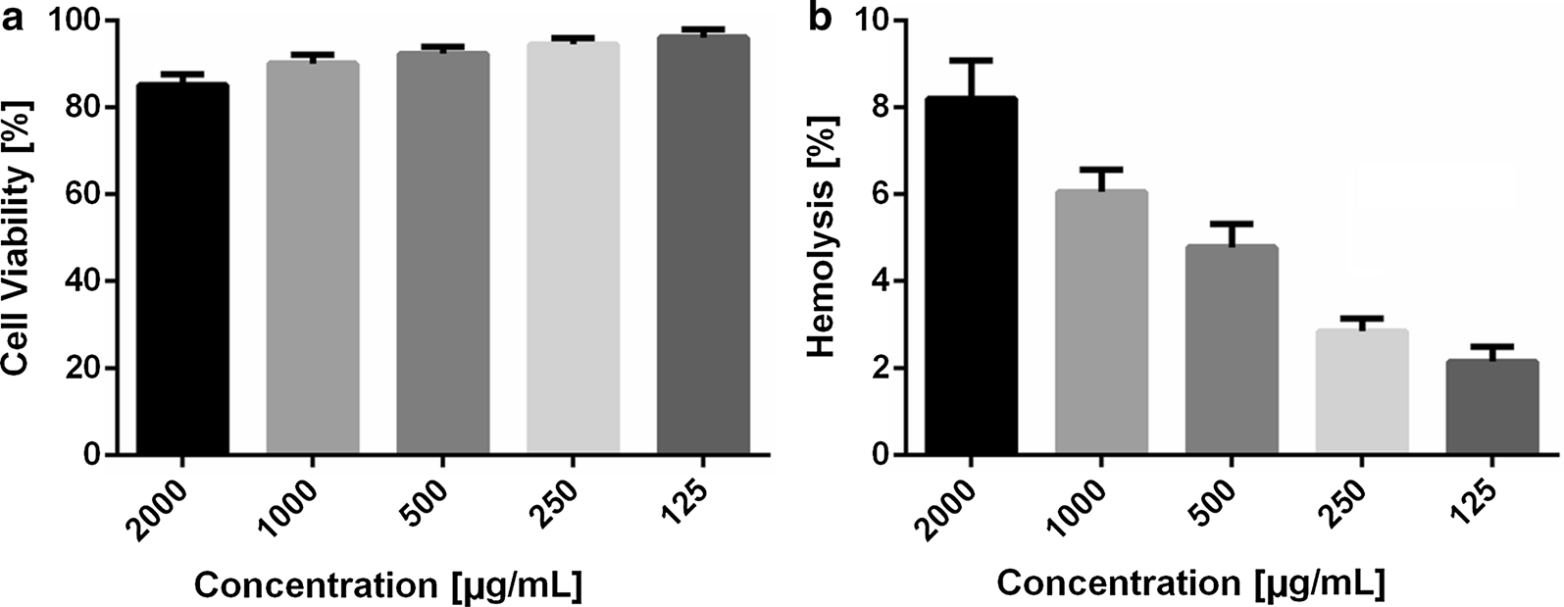
Cell viability of Vero cells after incubation for 24 h with AZM-SCSMs as a function of AZM concentration (**a**). Hemolysis behavior of AZM-SCSMs as a function of AZM concentration (**b**). n = 5 for all research

Cytotoxicity effect of AZM-SCSMs to normal cells was measured by MTT assay. As shown in Fig. 6a, AZM-SCSMs showed very low cytotoxicity for Vero cells, since the viabilities of Vero cells were all above 85% even at the highest concentration of 2000 μg/mL, which was much higher than that used for in vivo antibiofilm evaluation. Additionally, during antibacterial treatments in vivo, cytotoxicity to blood cells also needed to be considered. As illustrated in Fig. 6b, the hemolysis rate increased with the concentration of AZM-SCSMs increasing. However, the rate change was tolerable (≈10%) and no significant hemolytic behaviors were observed, suggesting the good hemocompatibility of AZM-SCSMs and the potential application in vivo.

### In vivo Antibiofilm evaluation of AZM-SCSMs

**Fig. 7:**
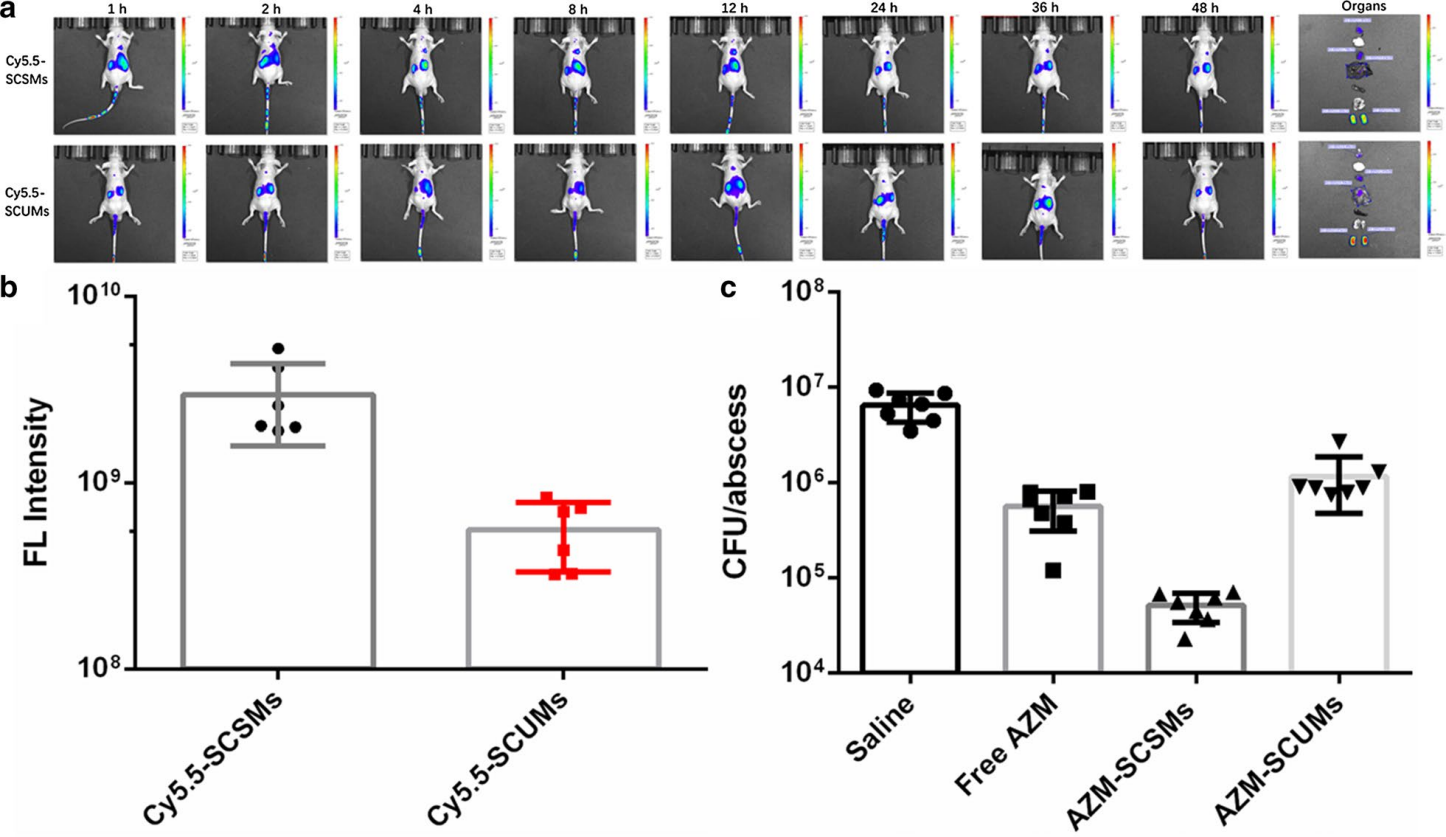
The in vivo non-invasive images of time-dependent whole body imaging and ex vivo optical images of abscess and organs (48 h) of bacterial infected BALB/c nude mice after *i.v.* injection of Cy5.5-SCSMs and Cy5.5-SCUMs (**a**). Quantitative statistics of abscess after treated with Cy5.5-SCSMs or Cy5.5-SCUMs for 48 h (**b**). Quantitative amounts of bacteria in abscess after receiving different treatments (Saline, free AZM, AZM-SCSMs and AZM-SCUMs) (**c**)

Inspired by the ideal antibiofilm effect of AZM-SCSMs, the practical applicability of AZM-SCSMs for in vivo antibiofilm applications was studied. Firstly, we would like to research if the SCSMs could be assimilated in the infected region. Cy5.5-SCSMs and Cy5.5-SCUMs were intravenously injected into the abscess-bearing BALB/c nude mice through the tail vein. As shown in Fig. 7a, the group of Cy5.5-SCSMs showed fairly strong fluorescence compared with Cy5.5-SCUMs treated group, and the residence time at the infected tissue was relative long, which indicated that positive charge reversal of SCSMs in weak acidic infected region was beneficial to effective gathering and long-term retention inside in vivo biofilm. The mean fluorescent intensity of the abscess in the group of Cy5.5-SCSMa was 5.3-fold as those of Cy5.5-SCUMs treated ones (Fig. 7b).

Further evaluation the in vivo anti-biofilm effects, the therapy began at day 2 after infection, and lasted for 3 days. At the end of treatment with AZM-SCSMs, without obvious inflammation was appeared on scarfskin or dermis, indicating the excellent bactericidal effects, but the severe inflammation and abscess occurred on the mice treated with saline and AZM-SCUMs, signifying bad results of killing bacteria. At day 5, the mice were sacrificed and abscess was collected for further analysis. AZM-SCUMs behaved relatively low antibacterial ability in vivo with 90% decrease of bacterial colony in abscess. Different from the in vitro anti-biofilm results, the group of free AZM indicated better antibacterial capability than the group of AZM-SCUMs (Fig. 7c). The main reason is that AZM could be fed to the infected site by phagocytes, increasing the AZM concentration in infected region. In contrast, the mice treated with AZM-SCSMs with 99.5% decrease of bacterial colony proved the greatest antibacterial effect.

## Conclusions

Recently, how to resolve the bacterial resistance in the clinical is a big challenge. Especially, the formation of biofilms complicates antibiotic treatment due to their protective effect on bacteria. Actually, the penetration of nanoparticles into biofilms is a significant and complex problem. By nanoparticles as carrier to deliver antibiotics achieving promoted biofilm penetration and reduced side effects is an effective strategy to defeat bacterial resistance. So as to address this problem, in this research, the acylhydrazone bond based micelles (SCSMs) were fabricated which can achieve simultaneous charge reversal in acidic biofilm microenvironment without destroying the micellar integrity. It is very important that the micelles could retain their integrity to facilitate drug-bacterium interactions. Further loaded AZM, AZM-SCSMs demonstrated enhanced biofilm penetration, excellent anti-biofilm efficiency both in vitro and in vivo. In conclusion, this strategy exhibited outstanding biofilm penetration and bactericidal capacity, and could be applicable for the delivery of other antimicrobial compounds.

## Supplementary Information

**Additional file 1.** The details of synthesis and characterization of the copolymers.

## Data Availability

All data generated or analysed during this study are included in this published article.
