# Supplementary material for: On-demand pH-sensitive surface charge-switchable polymeric micelles for targeting Pseudomonas aeruginosa biofilms development
Source: J Nanobiotechnology. 2021 Apr 9;19:99. doi: 10.1186/s12951-021-00845-0 (PMC8034112; doi:10.1186/s12951-021-00845-0)
Supplement: Supplementary file 1 — Additional file 1. The details of synthesis and characterization of the copolymers. [file 12951_2021_845_MOESM1_ESM.docx]

***Supplementary material of***

**On-Demand pH-sensitive Surface Charge-Switchable Polymeric Micelles for Targeting *Pseudomonas aeruginosa* Biofilms Development**

Xiangjun Chen^1^, Rong Guo^1^, Changrong Wang, Keke Li, Xinyu Jiang, Huayu He, Wei Hong^*^

*School of Pharmacy, Shandong New Drug Loading & Release Technology and Preparation Engineering Laboratory, Binzhou Medical University,* *346 Guanhai Road, Yantai, 264003, P. R. China.*

^*^*Corresponding author. Tel./ Fax.: +86-**0535-6913718*

*E-mail address:* [*hongwei_sy@* *bzmc.edu.cn*](mailto:hongwei_sy@163.com)

**1. Experimental Section**

**1.1** **Synthesis of PLA_5K_-PEI_2K_-hyd-mPEG_5K_**

PLA_5K_-COOH (1.0 g), DCC (1.2 eq) and NHS (1.2 eq) were completely dissolved into 10 mL chloroform, and the mixture solution was stirred for 2 h at room temperature. Then, 2 mL of PEI_2K_ chloroform solution was added, and the reaction was administrated at for another 24 h at room temperature. After reaction, the solvent was removed by rotary evaporation, and the crude product was redissolved with 5 mL of DMF. The DMF solution was dialyzed (molecular weight 3500) against Milli-Q water for 48 h, and then the dialysate was finally freeze-dried to obtain PLA_5K_-PEI_2K_.

0.5 g of mPEG_5K_-hyd-COOH was firstly dissolved in 10 mL of chloroform, and then DCC (1.2 eq) and NHS (1.2 eq) were added to dissolve completely. After reacting for 2 h, PLA_5K_-PEI_2K_ (1.0 eq.) was added, and the reaction was maintained for another 24 h at room temperature. At the end of reaction, the solvent was evaporated, and the product was redissolved with 5 mL of DMF. The DMF solution was transferred to dialysis bag (molecular weight 8000) and dialyzed with Milli-Q water for 48 h. Finally, the dialysate was collected and freeze-dried to obtain the product of PLA_5K_-PEI_2K_-hyd-mPEG_5K_. The detailed synthetic route was shown in Fig. S1.


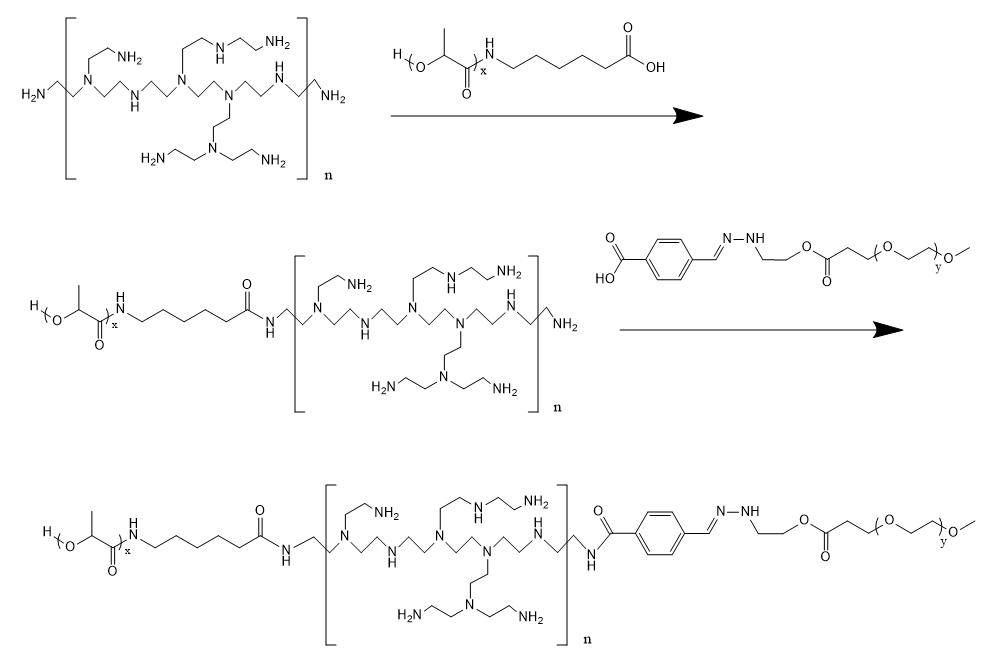


**Fig. S1** The detailed synthetic route of PLA_5K_-PEI_2K_-hyd-mPEG_5K_

**1.2 Synthesis of** **PLA_5K_-PEI_2K_-mPEG_5K_**

PLA_5K_-PEI_2K_-mPEG_5K_ was synthesized as the same procedure as PLA_5K_-PEI_2K_-hyd-mPEG_5K_ described above, except for using mPEG_5K_-COOH instead of mPEG_5K_-hyd-COOH.

The detailed synthetic route was shown in Fig. S2.


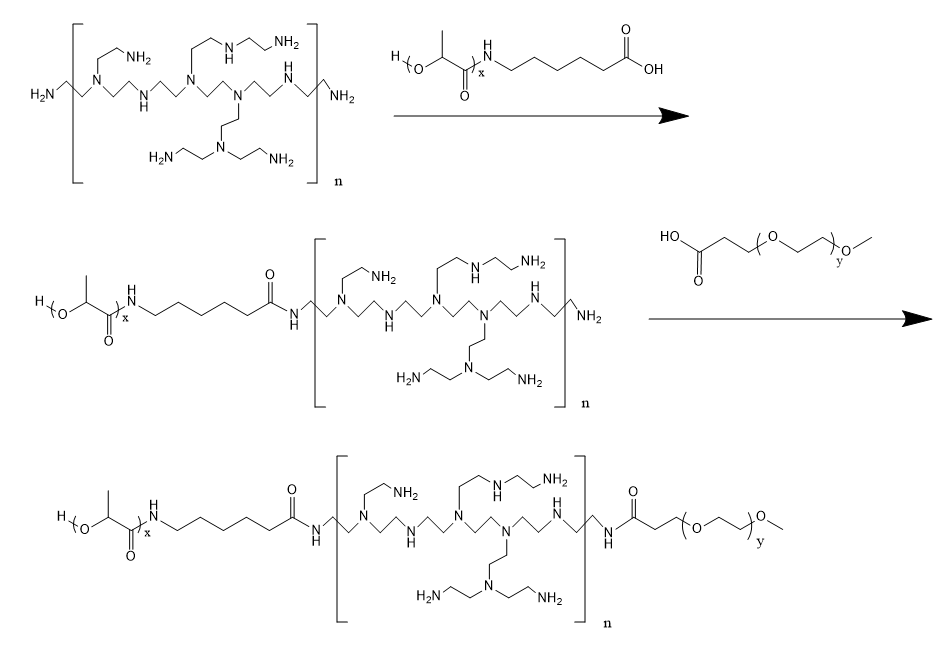


**Fig. S2** The detailed synthetic route of PLA_5K_-PEI_2K_-mPEG_5K_

**1.4 Characterization of copolymers**

The chemical structures of PLA_5K_-COOH, PLA_5K_-PEI_2K_, PLA_5K_-PEI_2K_-hyd-mPEG_5K_ and PLA_5K_-PEI_2K_-mPEG_5K_ were characterized by a Bruker DRX-600 NMR instrument at 600 MHz. The molecular weight distribution of PLA_5K_-COOH, PLA_5K_-PEI_2K,_ PLA_5K_-PEI_2K_-hyd-mPEG_5K_ and PLA_5K_-PEI_2K_-mPEG_5K_ were measured by a gel permeation chromatography (GPC) system. N,N-Dimethylformamide (DMF) was used as eluent with a flow rate of 1 mL/min at 40 °C.

**2. Results**

**2.1 Characterization of copolymers**

The ^1^H NMR spectra of final copolymers were shown in Fig. S3. All the chemical shifts were expressed in parts per million (δ) relative to the solvent signal. The ^1^H NMR spectrum (CDCl_3_) of PLA_5K_-COOH (Fig. S3A) showed the characteristic peaks at δ=5.21 ppm (-HO-CH-CO-) and δ=1.59 ppm (-CH_2_-CH_2_-NH_2_). The ^1^H NMR spectrum (CDCl_3_) of PLA_5K_-PEI_2K_ (Fig. S3B) showed the characteristic peaks of both PLA block and PEI block at δ=5.21 ppm (-HO-CH-CO-), δ= 2.73 ppm (-NH-CH_2_-CH_2_-NH-) and δ=1.59 ppm (-CH_2_-CH_2_-NH_2_), respectively. The ^1^H NMR spectrum (CDCl_3_) of final product PLA_5K_-PEI_2K_-hyd-mPEG_5K_ (Fig. S3C) showed peaks at δ=5.21 ppm (-HO-CH-CO-), δ=3.66 ppm (-O-CH_2_-CH_2_-OH), δ= 2.73 ppm (-NH-CH_2_-CH_2_-NH-), and δ=1.57 ppm (-CH_2_-CH_2_-NH_2_), which represented the characteristic peaks of PLA block, PEI block and mPEG block. The ^1^H NMR spectrums (CDCl_3_) of PLA_5K_-PEI_2K_-mPEG_5K_ (Fig. S3D) also showed the characteristic peaks of PLA block, PEI block and mPEG block, suggesting the copolymer was obtained.


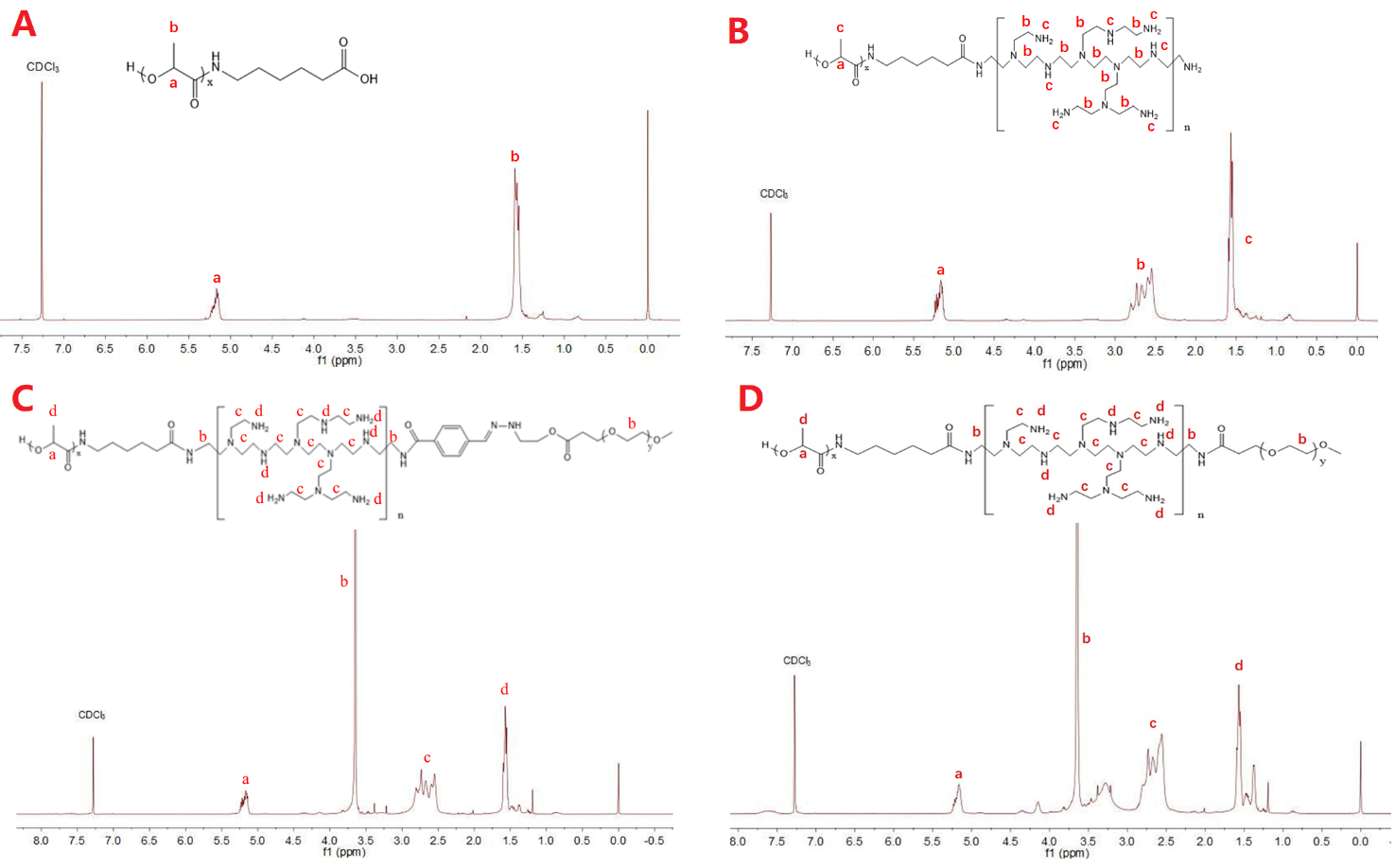


**Fig. S3** ^1^H NMR spectrum of PLA_5K_-COOH (A), PLA_5K_-PEI_2K_ (B), PLA_5K_-PEI_2K_-hyd-mPEG_5K_ (C) and PLA_5K_-PEI_2K_-mPEG_5K_ (D)

The GPC chromatograms of PLA_5K_-COOH, PLA_5K_-PEI_2K_, PLA_5K_-PEI_2K_-hyd-mPEG_5K_ and PLA_5K_-PEI_2K_-mPEG_5K_ were shown in Fig. S4. All the copolymers showed unimodal distribution with a polydispersity < 1.5. The shorter elution time of triblock copolymers of PLA_5K_-PEI_2K_-hyd-mPEG_5K_ and PLA_5K_-PEI_2K_-mPEG_5K_ than that of the homopolymer of PLA_5K_-COOH and diblock copolymer of PLA_5K_-PEI_2K_ indicated an increase in the molecular weight and the progress of the reaction. The number average molecular weight (M_n_) of PLA_5K_-COOH, PLA_5K_-PEI_2K_, PLA_5K_-PEI_2K_-hyd-mPEG_5K_ and PLA_5K_-PEI_2K_-mPEG_5K_ calculated by GPC was 4424, 6482, 11152 and 10686, respectively.


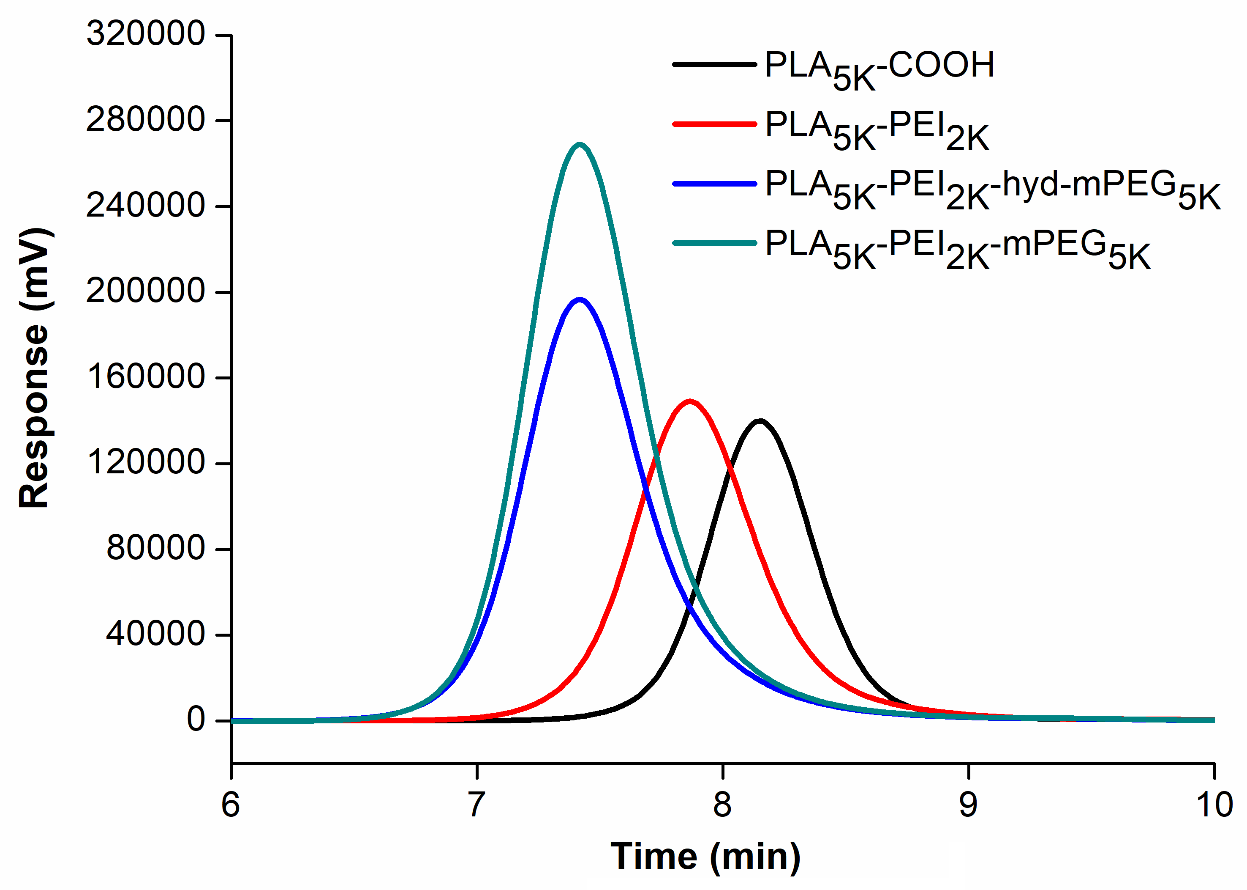


**Fig. S4** The GPC chromatograms of PLA_5K_-COOH, PLA_5K_-PEI_2K_, PLA_5K_-PEI_2K_-hyd-mPEG_5K_ and PLA_5K_-PEI_2K_-mPEG_5K_. The polydispersity (Ð) values of PLA_5K_-COOH, PLA_5K_-PEI_2K_, PLA_5K_-PEI_2K_-hyd-mPEG_5K_ and PLA_5K_-PEI_2K_-mPEG_5K_ were 1.18, 1.30, 1.37 and 1.34, respectively.
